# Long Term Retention of Gingival Sealing around Titanium Implants with CaCl_2_ Hydrothermal Treatment: A Rodent Study

**DOI:** 10.3390/jcm8101560

**Published:** 2019-09-29

**Authors:** Yasunori Ayukawa, Wakana Oshiro, Ikiru Atsuta, Akihiro Furuhashi, Ryosuke Kondo, Yohei Jinno, Kiyoshi Koyano

**Affiliations:** 1 Section of Implant and Rehabilitative Dentistry, Division of Oral Rehabilitation, Faculty of Dental Science, Kyushu University, Fukuoka 812-8282, Japan; 2 Division of Advanced Dental Devices and Therapeutics, Faculty of Dental Science, Kyushu University, Fukuoka 812-8282, Japan; 3 Department of Oral and Maxillofacial Surgery and Oral Medicine, Faculty of Odontology, Malmö University, Malmö S-20506, Sweden

**Keywords:** calcium hydrothermal treatment, dental implant, epithelial cell, titanium, down-growth

## Abstract

We previously reported that CaCl_2_ hydrothermal-treated (Ca-HT) titanium (Ti) implants induced a tight sealing at the interface between the implant and peri-implant epithelium (PIE) after implantation. However, it is not clear how long this improved epithelium sealing can be maintained. We subsequently investigated whether the positive effect of Ca-HT to promote sealing between the PIE and implant was sustained longer term. Maxillary molars were extracted from rats and replaced with either Ca-HT implants (Ca-HT group), distilled water-HT implants (DW-HT group) or non-treated implants (control group). After 16 weeks, the majority of implants in the Ca-HT group remained at the maxillary with no apical extension of the PIE. Conversely, half the number of control implants was lost following down-growth of the PIE. The effect of Ca-HT on migration and proliferation of rat oral epithelial cells (OECs) was also investigated. In OECs cultured on Ca-HT Ti plates, protein expression in relation to cell migration decreased, and proliferation was higher than other groups. Surface analysis indicated HT enhanced the formation of surface TiO_2_ layer without altering surface topography. Consequently, Ca-HT of Ti reduced PIE down-growth via tight epithelial attachment to the surface, which may enhance implant capability for a longer time post-implantation.

## 1. Introduction

Dental implants are reported to have high success rate but peri-implantitis is one of the most important reason for the failure of implants. Peri-implantitis is initiated by the deterioration of implant-gingival adhesion associated with inflammatory cells [1], with the adhesion of microorganisms being a prerequisite for infection. To prevent peri-implantitis, various materials, topographies and chemical modification have been investigated. Utilizing materials that improve the peri-implant soft tissue sealing could reduce the risk of infection, which consequently may improve implant longevity. As the example of surface modification of dental implants, hydroxyapatite (HA) coating is known to optimize osseointegration [2]. HA-coated implants can function successfully for more than 5 years [3]. To enhance soft tissue attachment to the transmucosal part of the implant, grooving on the surface has been reported to promote soft tissue attachment, with groove size being critical to success [4], however, the use of a textured surface increases the risk of plaque accumulation [5]. 

In particular, epithelial down-growth around dental implants destabilizes the soft tissue-implant interface by providing a route for invasion of external pathogens, which may be a reason for implant failure. Periodontal pocket formation provides a moist, warm and anaerobic environment for bacterial colonization, with mechanical cleaning difficulties. Although the epithelial tissue can go down through damaged connective tissue, healthy collagenous matrix can stop this migration [6]. Down-growth occurs as a direct result of the normal wound healing signal cascade and the ‘free edge effect’, whereby growth factors and the absence of neighboring cells act as signals for proliferation and migration of the wound edge epithelial cells to re-establish layer continuity [7,8]. For implant success, it is crucial to prevent epithelial down-growth with implant surface to promote epithelial cell attachment. To prevent epithelial down-growth, platform switching is introduced. In this concept, abutments of narrower diameter are used with implant of wider diameter. This can prevent epithelial down-growth to some extent [9], but narrow abutment sometimes cause an increase in stress concentration to certain components [10]. Some possible surface modification procedures such as ultraviolet or non-thermal plasma irradiation have also been reported [11] to enhance cell attachment.

In our previous study using rodent, both the integration of soft tissue cells with titanium and the epithelial sealing against foreign body penetration were improved at 4 weeks after implantation when the titanium implant was hydrothermally-treated with calcium chloride (Ca-HT) [12]. However, the longevity of the effect of HT with calcium remains to be elucidated because long-term enhancement of epithelial sealing is clinically expected. The null hypothesis of the present study is that Ca-HT has no impact for the prevention of epithelial down-growth to maintain the structure using a rat implant model for a longer period (16 weeks). 

## 2. Materials and Methods

### 2.1. Ti implants and Plates

**Figure 1:**
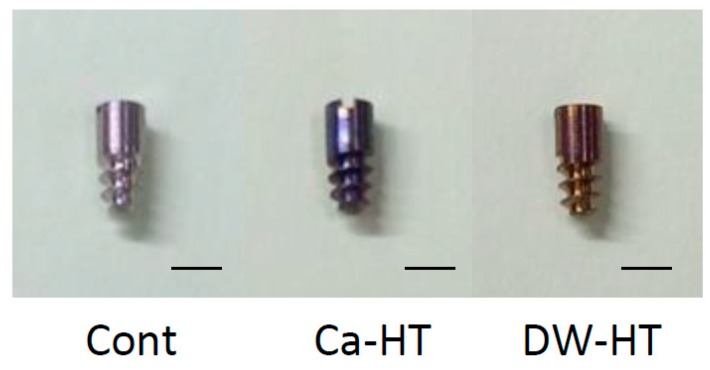
Experimental implants: titanium implant without any treatment (Control group: Cont), CaCl_2_-hydrothermally treated implant (Ca-HT) and distilled water-hydrothermally treated implant (DW-HT) (bar = 2 mm).

Commercially available, mirror-polished commercially grade 1 pure titanium plates (15 mm in diameter, 1 mm in thickness (KS40, Kobelco, Kobe, Japan)) and experimental implant bodies (4.5 mm in length, 2 mm in diameter; Figure 1) (Skyblue, Fukuoka, Japan) were used.

Implants in the control group (Cont) received no treatment. Implants in the HT with distilled water (DW) group (DW-HT) received a hydrothermal treatment in DW at 200 °C in a pressure pot for 24 h. Implants in the CaCl_2_ treatment group (Ca-HT) received a hydrothermal treatment with 10 mmol/L CaCl_2_ at 200 °C in a pressure pot for 24 h [13,14]. After treatment, all implants were rinsed with DW and stored under a vacuum to prevent surface contamination. Surface Ra was in the range of 0.20‒0.22 μm (Table 1).

### 2.2. Surface Characterization with Thin-Film X-ray Diffractometry (TF-XRD)

The composition of each specimen was determined using an X-ray diffractometer (SmartLab, Rigaku, Tokyo, Japan) operated at 40 kV and 30 mA. The diffraction angle was continuously scanned from 20°–90° in 2 theta.

### 2.3. Oral Implantation

**Figure 2:**
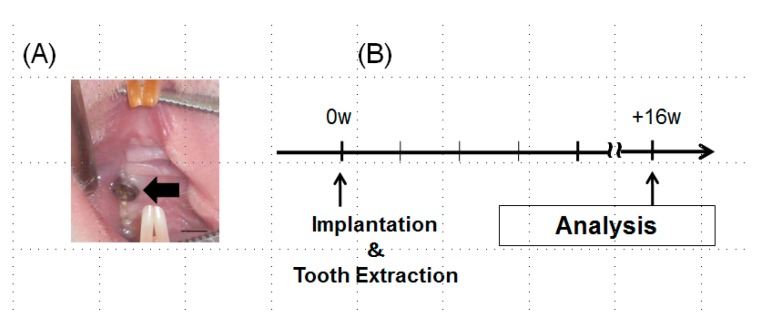
(**A**) An intraoral view of the experimental implant. No significant inflammatory signs were observed in the oral mucosa surrounding the implants (arrow) (bar = 1mm). (**B**) Experimental protocol for the in vivo study. Peri-implant epithelial tissue structure was observed after 16 weeks of implantation. To observe down-growth of peri-implant epithelium and the distribution of laminin-332, tissues were obtained immediately after the sacrifice and processed. For horseradish peroxidase (HRP) penetration study, thirty minutes before sacrifice, HRP was topically applied.

Rats (6-week-old Wistar rats, 27 males weighing 120–150 g) were treated in accordance with the NIH Guide for the Care and Use of Laboratory Animals, after approval of the research protocol by the ethical committee of Kyushu University (approval number: A24-237-0). The Animal Research: Reporting In Vivo Experiments (ARRIVE) guidelines for the execution and submission of studies in animals were followed. Details of the implant placement were described in the previous manuscripts [15,16]. In brief, under systemic and local anesthesia, the extraction of the maxillary right first molar of rat was done and the titanium screws were inserted into the extraction socket (Figure 2A). Following surgery, buprenorphine (0.05 mg/kg, intramuscular injection) was injected as an analgesic. Animals were euthanized 16 weeks after the implantation (Figure 2B). 

### 2.4. Topical Application of Horseradish Peroxidase

Horseradish peroxidase (HRP, 50 mg/mL, type 11, molecular weight 40,000 Da; Sigma Aldrich, St. Louis, MO, USA) was applied around the implant at 16 weeks after implantation to evaluate the secureness of epithelial sealing. The procedure for topical application of HRP was similar to that reported previously [15]. Under systemic anesthesia, cotton balls were immersed with HRP (50 mg/mL) and put at the gingival margin around the implant as gently as possible to avoid the damage at the interface between implant and gingiva. HRP solution was further dropped into the cotton balls every 10 min for one hour. 

### 2.5. Tissue Preparation

Tissues were prepared as described previously [17]. After 16 weeks, rats were euthanized and perfusion fixation was done with 4% paraformaldehyde (pH 7.4). The maxillae were dissected and decalcified in 5% EDTA for 4 days at 4 °C. Peri-implant or periodontal oral mucosa was carefully peeled from the bone, implant or tooth. All specimens were cut into 10-µm palate-buccal sections with a cryostat at −20 °C.

### 2.6. HRP Histochemistry 

Several 10-μm-thick sections were washed with 0.01M PBS and incubated in 3,3’-Diaminobenzidine tetrahydrochloride (DAB, Dojin Laboratories, Kumamoto, Japan) medium containing 0.02% DAB and 0.006% H_2_O_2_ in 0.05 M Tris-HCl buffer, pH 7.6, for 5 min at room temperature. For controls, additional sections were incubated in DAB without H_2_O_2_. All sections were observed with a light microscope [18].

### 2.7. Immunohistochemistry

As described previously, an avidin-biotin complex (ABC) procedure was employed for immunohistochemical staining [17]. In brief, sections were incubated in 10% normal goat serum for 30 min, then further incubated overnight at 4 °C with rabbit polyclonal anti-rat laminin 332 (Ln) γ2 chain antibody (1:100 dilution of clone 2778, kindly provided by Dr. Vito Quaranta, Scripps Research Institute, La Jolla, CA, USA). Afterward, sections were exposed to biotin-conjugated goat anti-rabbit IgG (1:200) for 45 min, and finally with the ABC reagent (1:100) for 60 min (Vectastain ABC, Vector Laboratories, Burlingame, CA, USA). Immunoreaction was visualized using 0.02% DAB and 0.006% H_2_O_2_, and counterstained with hematoxylin.

### 2.8. Quantification of HRP Penetration and Down-Growth

Five frozen sections were selected from the buccal side of peri-implant epithelium (PIE) and used for the linear measurements, with the landmarks to measure the vertical distance between the top of the gingival edge and bottom of the PIE.

### 2.9. Cell Culture

**Figure 3:**
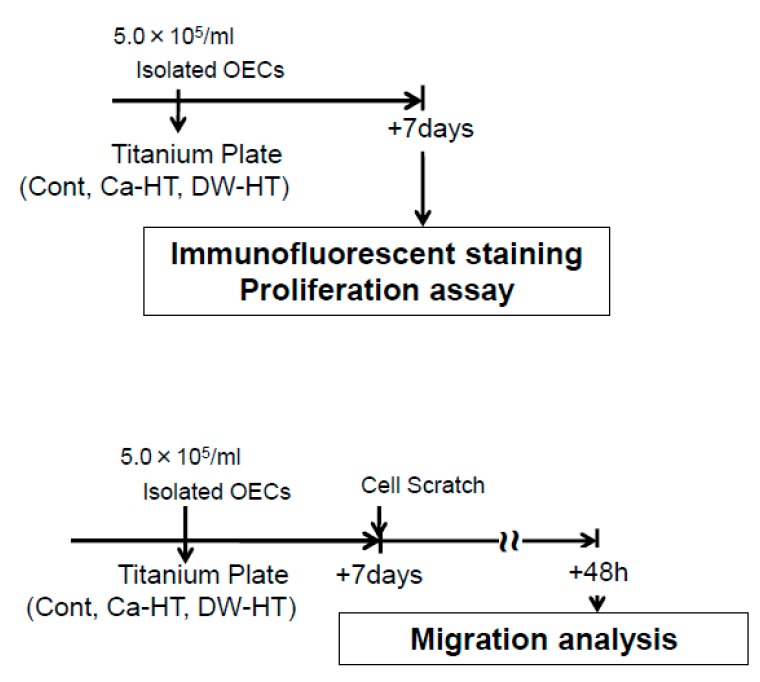
Experimental protocol for the in *vitro* study. Rat oral epithelial cells (OECs) were analyzed for changes in cell morphology 7 days after seeding OECs on Cont, hydrothermally-treated titanium in CaCl_2_ solution (Ca-HT) or hydrothermally-treated titanium in distilled water (DW-HT) groups. For migration assay, confluent monolayers of OECs were scratched with a cell scraper and further cultured for 48 h. Afterward, cell number migrated into scratch area was counted.

Oral epithelial cells (OEC) were cultured as reported in the previous report [19]. Briefly, oral mucosa collected from 4-day-old Wistar rats was incubated with dispase (1×10^3^ IU/mL) for 12 h at 4 °C. The oral epithelium was then removed and dispersed by pipetting 10 times and seeded onto Ti plates. OECs were cultured in defined keratinocyte serum free medium (DK-SFM; Invitrogen, Grand Island, NY, USA) and gentamycin (50 μg/mL) in a humidified atmosphere of 5% CO_2_ at 37 °C. All culture experimental data were analyzed 7 days after the cell seeding (Figure 3).

### 2.10. Immunofluorescence Staining for Adhesion Proteins

OECs were fixed in 4% formaldehyde for 10 min and permeated in 0.5% Triton X-100 (Novocastra Laboratories, Newcastle-upon-Tyne, UK) for 3 min. OECs were then incubated overnight at 4 °C with a polyclonal rabbit anti-rat E-cadherin antibody (Santa Cruz Biotechnology, Santa Cruz, CA, USA; 1:100) or polyclonal mouse anti-rat Integrin α3 (In-α3) antibody (Santa Cruz Biotechnology; 1:100). Cells were further incubated with fluorescein isothiocyanate (FITC)-labeled secondary antibody (Chemicon International, Billerica, MA, USA; 1:100 dilution) for 2 h at 37 °C. Actin filaments were visualized with tetramethylrhodamine -labeled phalloidin (Sigma-Aldrich; 1:100) for 1 h at 37 °C. Nuclear stain was done using 4′6-diamidino-2-phenylindole (DAPI, VECTASHIELD mounting medium, Vector Laboratories). Cells were observed using a fluorescence microscope (BZ-9000, Keyence, Osaka, Japan).

### 2.11. Western Blot Analysis

OECs seeded onto each substrate were lysed in lysis buffer and centrifuged at 13,000 g for 10 min. Proteins were separated on 7.5% polyacrylamide gel, transferred to immun-Blot PVDF membranes^®^ (Bio-Rad Laboratories, Hercules, CA, USA) and immunoblotted with polyclonal antibodies (rabbit anti-rat Ln-γ2 chain (clone 1967; provided by Dr. Vito Quaranta; 1:100), rabbit anti-rat In-β4 (1:100), or mouse anti-rat plectin (1:100)) for 24 h at 4 °C. Blots were then incubated with HRP-labeled goat anti-rabbit IgG secondary antibody (Amersham Biosciences, Little Chalfont, UK) for an hour at room temperature. Pictures were taken using a chemiluminescence system (Amersham Biosciences).

### 2.12. Proliferation Assay

A cell proliferation kit (Molecular Probes, Eugene, OR, USA) was employed to detect cell proliferation. Briefly, OECs were exposed to 5-bromo-2′-deoxyuridine (BrdU) in culture medium for 1 h and then fixed in 4% formaldehyde for 10 min. The presence of BrdU was visualized using mouse anti-BrdU monoclonal antibody (1:100) with FITC-labeled anti-mouse IgG (Chemicon International; 1:100).

### 2.13. Scratch Assay

Scratch assays on Ti plates were performed as a model for wound closure [16]. Confluent monolayers of OECs were removed using a scraper and cultured for 48 h. Cells at the edge of the scratch were observed by immunofluorescence using antibodies against actin and chemical fluorescence using carboxyfluorescein succinimidyl ester for cell visualization.

### 2.14. Statistical Analysis

Data are expressed as means ± SD. One-way analysis of variance with Tukey’s post hoc test was performed. The survival rates of experimental implants for each time point were analyzed by the log-rank test. *P* < 0.05 were considered to be statistically significant.

## 3. Results

### 3.1. Surface Characterization with TF-XRD 

**Figure 4:**
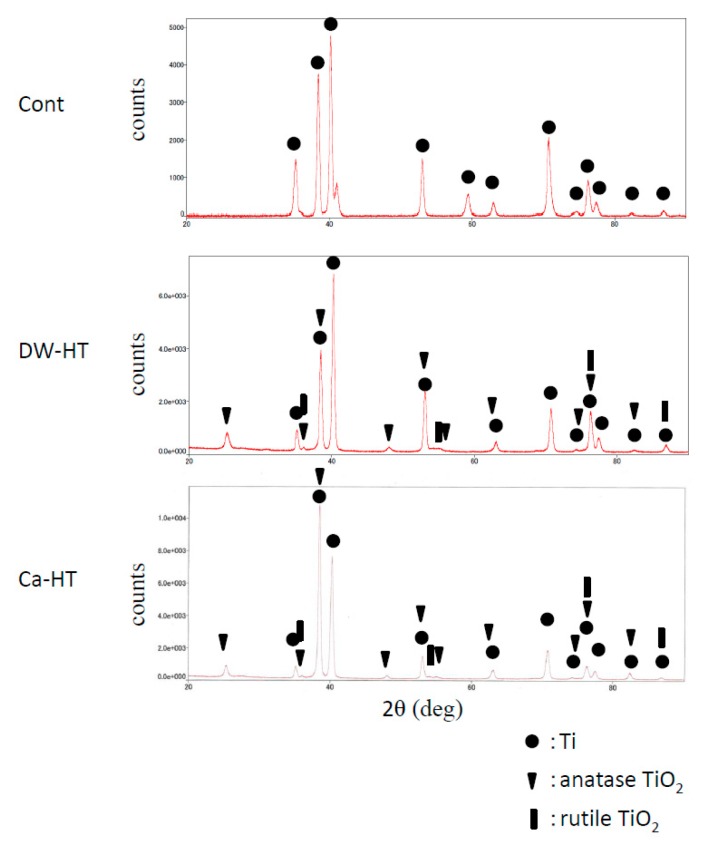
TF-XRD patterns of Cont, DW-HT and Ca-HT. TiO_2_ peaks were indicated in both HT groups.

Results of TF-XRD patterns of the disks used in the present study indicated that Cont represented titanium peaks and no TiO_2_ peaks were observed. Unlike the result of Cont, both hydrothermal groups represented TiO_2_ peaks, as well as Ti peaks (Figure 4).

### 3.2. Implant Survival Rate in Rat Oral Environment.

**Figure 5:**
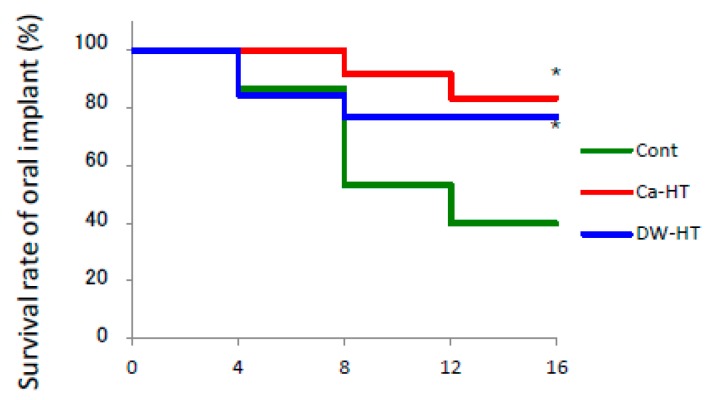
Survival rate at 4–16 weeks. The rate in the Cont group was dramatically decreased after 8 weeks. The Ca-HT and DW-HT groups were similar in change, but in both cases reductions were slower than in the Cont group. Log-rank test. * *P* < 0.05 vs. Cont.

The survival rate of the Cont group dramatically decreased to less than 60% after 16 weeks (Figure 5). There was no statistically significant difference between the survival rate of the Ca-HT and DW-HT groups and survival reductions were much slower than in the Cont group.

### 3.3. Down-Growth of PIE and Distribution of Laminin-332 around the Experimental Implants 

**Figure 6:**
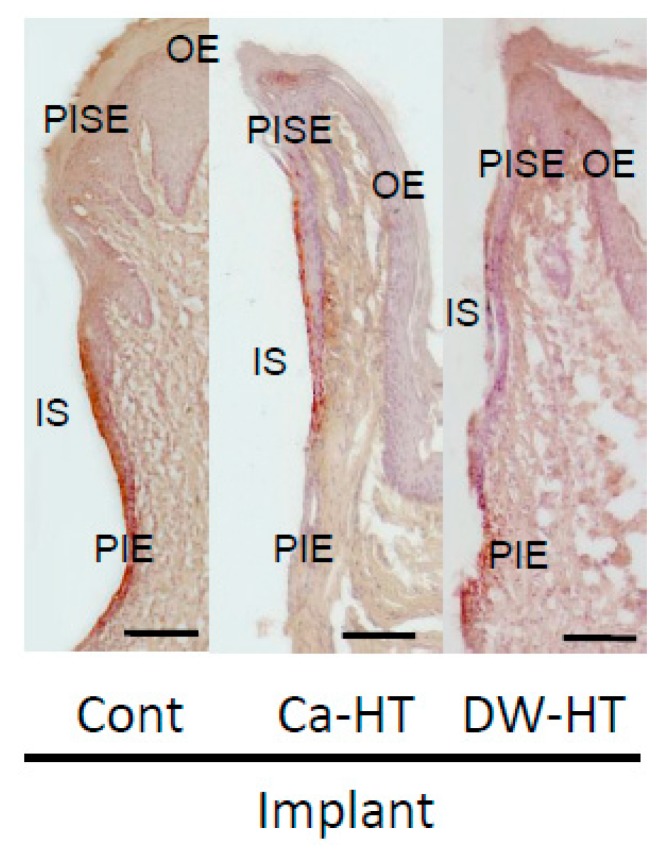
Light micrographs of Ln localization in the oral mucosa around the implants after 16 weeks. The localization of Ln was seen as a band along the implant-PIE interface. Localization of Ln was restricted at the interface between implant and PIE in Ca-HT group. (IS, implant space; OE, oral epithelium; PISE, peri-implant sulcular epithelium; PIE, peri-implant epithelium. Hematoxylin staining. Bar = 100 μm.

In Ca-HT group, the localization of Ln was limited, along the implant surface. Conversely, in the groups other than Ca-HT, Ln-immunoreactivity was diffusely scattered in the connective tissue from the apical area of the PIE. Interestingly, in Cont and DW-HT groups, but not in Ca-HT group, apical down growth of the PIE was observed at 16 weeks (Figure 6).

### 3.4. Penetration of HRP in the Peri-Implant Soft Tissue

**Figure 7:**
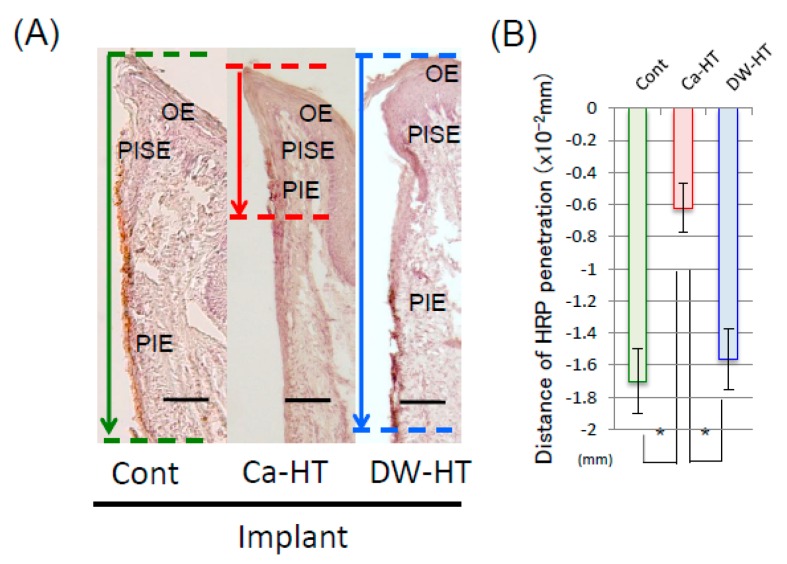
(**A**) Light micrographs of the localization of HRP around the implant. The localization of HRP was restricted in the upper PIE in the Ca-HT group. In other groups, HRP was penetrated more deeply and widely than the Ca-HT group. Bar = 100 μm. (**B**) Depth of HRP penetration. Each bar represents the mean ± SD. * *P* < 0.05 vs. Ca-HT.

In the apical portion of the PIE of the Cont and DW-HT groups, HRP was penetrated into deeper portions on the surface of experimental implant. Conversely, HRP reactions around the apical area disappeared by the end of the PIE in the Ca-HT group (Figure 7).

### 3.5. Expression of E-cadherin, In-α3 and Filamentous Actin in OECs

In regard to the In-α3 subunit, numerous and punctate immune-reactivity was present throughout the OEC cytoplasm in the Cont and DW-HT groups. 

**Figure 8:**
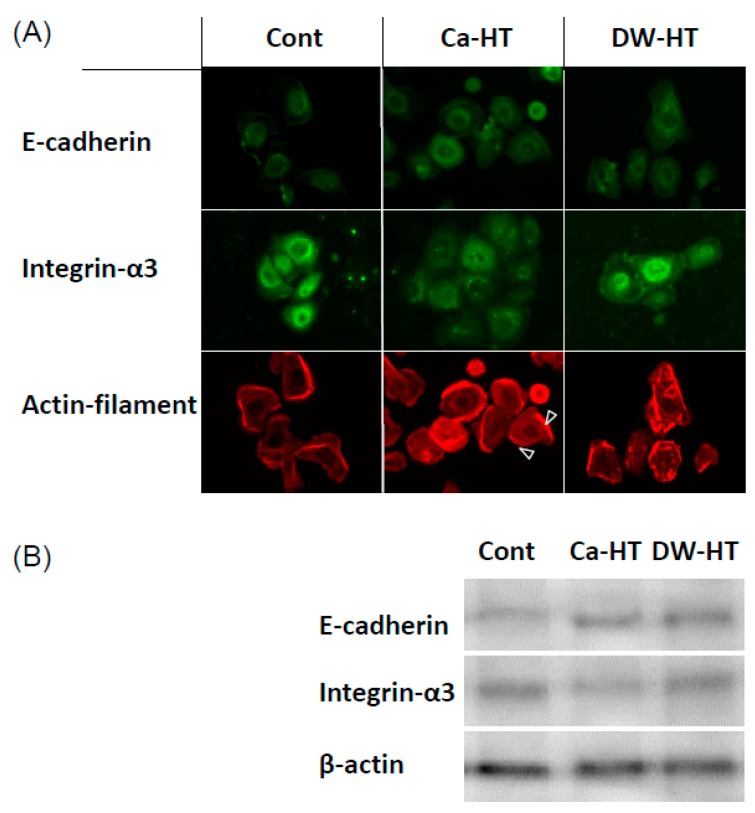
(**A**) Localization of cell migration-related proteins in OECs. Cells seeded onto Ca-HT represented well-organized actin ring. Numerous and punctate immune-reactivity of In-α3 was present throughout the OEC cytoplasm in the Cont and DW-HT groups. The Cont group had a similar distribution of E-cadherin in cells to both the Ca-HT and DW-HT groups. (**B**) Western blot analysis of adhesion proteins. Expression levels of In-α3 in the cells grown on the Ca-HT group were slightly lower compared with the Cont and DW-HT groups.

The Cont group had a similar distribution of E-cadherin in cells to both the Ca-HT and DW-HT groups. These immunoreactivities were seen at the peri-nuclear region in the OECs. Actin filaments were interrupted and/or less developed in the cells seeded onto the Cont and DW-HT groups. Western blotting showed that the expression levels of In-α3 in the cells grown on the Ca-HT group were slightly lower than those in other groups. These results are consistent with the immunofluorescence findings (Figure 8).

### 3.6. OEC Migration and Proliferation

**Figure 9:**
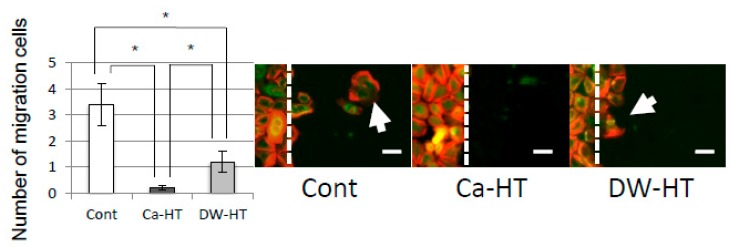
Scratch assay. (Left) The number of migrating cells on each substratum. Bars represent the mean ± SD. * *P* < 0.05. (Right) More cells (arrows) in both control and DW-HT groups were migrated into the scratched area (right side of the dotted line) than those in Ca-HT group. Bar = 20 μm.

At 48 h after the scratch, cells at this ‘leading edge’ had spread into the scratched area. In the Ca-HT group, this migration was remarkably inhibited (Figure 9). 

**Figure 10:**
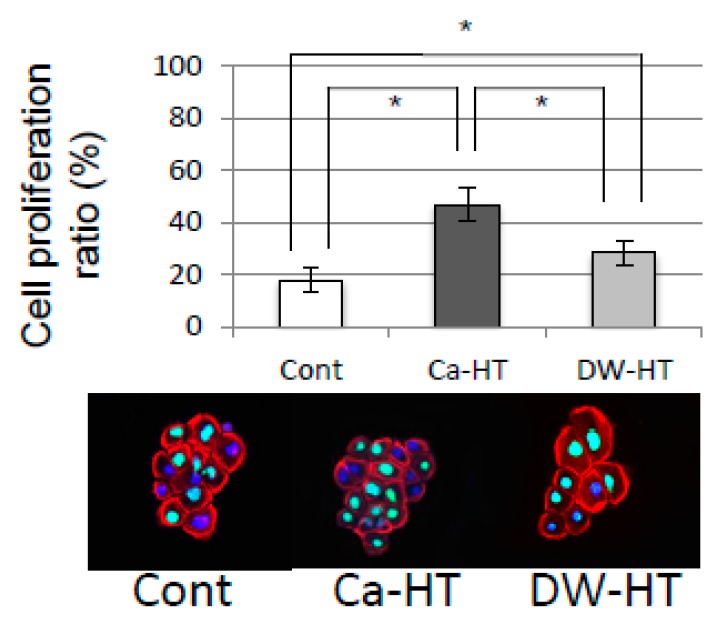
Cell proliferation assay. The percentages of OECs proliferating on Ca-HT, DW-HT and Cont were significantly different among the groups. Bars represent the mean ± SD. * *P* < 0.05.

Figure 10 indicated that cell proliferation ratio was highest in the Ca-HT group. The DW-HT group displayed significantly higher in cell proliferation than Cont group.

## 4. Discussion 

The present study was conducted to verify following null hypothesis that Ca-HT has no impact for the prevention of epithelial down-growth to maintain the structure using a rat implant model for a longer period (16 weeks). For this purpose, a rat oral implantation model was used in the present study. This model has been used for more than ten years. Using these original models, the similarity between the junctional epithelium (JE) and PIE has been shown at the point of construction and function [15]. Additionally, differences have also been shown in the strength of sealing around the tooth or implant [17]. At 4 weeks post-implantation, Ln-positive staining was apparent only in the lower potion on the experimental implant, while around the Nt, the Ln-immunoreactivity extended into the upper portion. These previous data showed that our rat model was effective in evaluating the soft tissue attitude toward implanted material. Indeed, 4 weeks post-implantation, the survival rate was over 80%. However, the rate was dramatically decreased below 50% after 16 weeks. We suggest three reasons for this reduction: (1) in this model, it is hard to control the unexpected stimulation on the rat implant; (2) the implant body penetrates into the maxillary sinus and is not covered by cortical bone, because the experimental implant is too large for the rat oral cavity; and (3) the implant with machined surface has integrated weaker with bone than commonly-used texture-surfaced implant. There was no difference in survival rate for both the Ca-HT and DW-HT groups from 4 to 16 weeks, and reductions were much slower than in the Cont group.

After 16 weeks, we observed that the experimental implant models exhibited apical down-growth of the PIE. Furthermore, a marked positive area of Ln at all surfaces of the PIE was observed in the Ca-HT group and at the bottom of the PIE in the Cont and DW-HT groups. The distance of PIE migration on the Ca-HT implants was shorter than on the Cont and DW-HT implants, consistent with the in vitro data. Additionally, these results showed that the Cont and DW-HT implants might easily cause deep pockets, and have a high risk of leading to implant failure. We speculate that apical movement, as well as soft tissue integration between the peri-implant mucosa and experimental implant can be prevented by Ca-HT. Furthermore, limited expression of Ln in the Cont and DW-HT implants showed much weaker sealing of the peri-implant soft-tissue than the Ca-HT implants. The PIE with weak OEC adhesion to the Ti surface on the Cont implants seemed unstable as described above, and was more inclined to migrate. 

The Ca-HT group had a significantly larger number of OECs than the DW-HT group. In our previous studies, the presence of Ca on the Ti surface effectively contributed to greater attachment of OECs. This result is consistent with the view that an enhancement of the cell activity by divalent cations, such as Ca or Mg ions, promotes initial epithelial cell attachment to a negatively charged substrate [13]. Therefore, OEC migration was inhibited on the Ti plates in the Ca-HT group, as determined by the scratch assay (Figure 9) and proliferation was promoted, as determined by the Brd-U assay (Figure 10). Additionally, immunofluorescence observed that expressions of some proteins related to migration were stronger in the Cont and DW-HT groups than in the Ca-HT group (Figure 8). In-α3β1 activates migration of epithelium cells. Therefore, down-growth of the PIE along the implant might be extended. However, we found Inβ4 was more strongly expressed in the Ca-HT group than in the other groups. In-α6β4 regulates adhesion of epithelial cells. Besides this, down-growth of the PIE was not observed in the Ca-HT group.

Wound closure speed in the scratch assay is affected by both the migration of outermost cells and the proliferation of the following cells [20]. In the present study, OECs on Ti samples in the DW-HT and Cont groups had a greater number of cells, moving from the border of the ‘wound’ into the denuded area, than on the Ca-HT group. This result is consistent with the view that cell-substrate attachment is important for subsequent cell proliferation [21]. We therefore suggest that the strong adhesion of OECs to Ca ions on the treated Ti may block cell migration by preventing extension of the cells that are important for cell migration [22]. Ca ions may not only act as a mediator between the adhesion proteins and the surface of Ti with negative charge, but also activate the fibroblast growth factor (FGF) pathway [23]. FGF is expressed by epithelial cells and mediates cell proliferation, migration and cell-to-cell communication [24] and is activated by heparan sulfate proteoglycan (HSPG) [23]. Additionally, the high affinity of heparin/heparan sulfate with FGF receptors requires Ca ions at physiological concentrations [24]. Therefore, our results reflect the higher adhesion ability of OECs and expression of adhesion proteins, including In-β4, plectin and Ln, on Ca-HT, than those on DW-HT.

Additionally, strong adhesion to the substrate is also one of the most important factors for cell proliferation [25]. Here, we observed increased OEC proliferation on the Ti plates in the Ca-HT group. Differences in cell activation, including proliferation and migration, between the Ca-HT and DW-HT groups, may be caused by the cell adhesion levels which are affected by Ca ions on the CaCl_2_ hydrothermal-treated Ti. 

Recent literatures indicated that oral implants are distinguished as foreign bodies by immune system. As a result, oral implants evoke a mild immunoreaction. And implants survive in the body in a delicate positive immune balance based on the individual host immune system [26,27]. Titanium ion may be a possible factor to disrupt this balance. It was reported in the previous study that Ti (IV) ion enhanced the reactivity of T lymphocyte and deviation and as a result, it might evoke the inflammatory side effects of titanium implants seen in patients [28]. On the other hand, TiO_2_ nanoparticles were reported to cause inflammation partly due to the change in several immune response relevant signaling pathways [29], but passive oxide film of TiO_2_ surface is basically stable in body and plays a crucial role for the excellent biocompatibility of titanium [30,31]. However, since surface oxide film of pure titanium is very thin (approximately 4 nm), this oxide layer is easily scratched off from titanium substratum by some factors such as mechanical damage, and subsequent release of titanium ion from non-protected pure titanium surface is possible. Actually, the release of titanium ion from implanted material has been reported [32,33]. Besides, commercially-available recent oral implants have surface with nano-rough topography, and nanoparticles may be easily detached from the implant surface, which may evoke immunoreaction [29]. The result of TF-XRD in this study indicated that hydrothermal treatment enhanced the formation of surface oxide layer in comparison with control pure titanium. This may reduce the risk of the release of titanium ion. Furthermore, since hydrothermal treatment hardly alters the surface topography, this treatment may also prevent the release of nanoparticle from the titanium surface. 

As reported previously, Ca-HT enhanced the bone-titanium contact [14]. Surface calcium may play a role in this phenomenon. Our another report using X-ray photoelectron spectroscopy indicated that calcium actually presented on the surface of titanium [13]. In our TF-XRD study, however, no calcium-related crystal structure was observed. This may denote that no intermetallic compounds such as calcium titanate are formed with Ca-HT. Actual coupling scheme between calcium and titanium after Ca-HT remains to be elucidated, but the bonding between calcium and titanium seems to be weak and calcium may be easily released from the surface of titanium. Nevertheless, epithelial sealing capability of Ca-HT still remained even after the 16 weeks of implantation and survival rate of implant in rat oral cavity was significantly higher than other groups. 

A lot of attempts have been reported to enhance soft tissue integration with abutment. For example, using abutment with smaller diameter than the diameter of the implant body, so-called platform-switching, is proved to prevent epithelial down-growth and foreign body penetration [9]. This is now used in the clinical situation. But using smaller diameter abutment was reported to cause the stress concentration to certain components [10]. Other several attempts such as ultraviolet or non-thermal plasma irradiation were proposed to enhance cell attachment onto titanium [11]. These procedures have not reported the long-term stability of their effects. In the present study, we can propose Ca-HT as another procedure to enhance soft tissue integration with titanium, using relatively inexpensive equipment. 

The limitation of this study is that we employed rat oral implantation model. Healing capability and the defense against infection of rat may be stronger than human. In contrast, both plaque control and the control of occlusal force were not provided. As a result, gingival reaction toward the implanted material in rat may be slightly different from human. Other limitation is that we used implants with machined surface because we would like to eliminate other factors than hydrothermal treatment. And we employed one-piece implant incorporating both the implant body and an integral fixed abutment in one piece. Both machine-surfaced implant body and one-piece implants are less popular in the clinical situation. Since rat oral cavity is very small, two-piece implant is difficult to use. But rat oral implantation model is well-established and using this model, we could reduce the use of larger animals.

## 5. Conclusions

The present data suggest that activated cell attachment by CaCl_2_ hydrothermal treatment can improve the adhesion of PIE cells to the Ti surface of titanium implants and may create a secure seal at the epithelium-implant interface and reducing epithelial down-growth, even after 16 weeks of implantation into rat maxillae. Insufficient adhesion of soft tissues can lead to the formation of deep peri-implant pockets, raising the likelihood of periodontal complications. In addition, this procedure enhanced the formation of surface oxide layer without altering surface topography. This approach may have a potential to enhance long-term implant stabilization. As a result, the null hypothesis “Ca-HT has no impact for the prevention of epithelial down-growth to maintain the structure using rat implant model for 16 week” was rejected within the limitations of the present study.

## Figures and Tables

**Table 1:** Characteristics of culture disks and experimental implants.

	Material	Surface	Surface roughness (Ra, μm)	Dimension
Implant for animal study	Pure titanium Japan Industrial Standards Class 1 (equivalent for ASTM grade I)	Machined	0.438	screw shape 2 mm in diameter 4.5 mm in length (2.5 mm transmucosal + 2 mm intrabony)
Disk for culture study	Pure titanium Japan Industrial Standards Class 1 (equivalent for ASTM grade I)	Mirror-polished	0.20–0.22	15 mm × 1 mm (diameter × thickness)
